# Cheyne Hospital for Sick and Incurable Children, Cheyne Walk, Chelsea

**Published:** 1892-12-31

**Authors:** Reginald Blunt

**Affiliations:** Secretary


### EDITOR'S LETTER-BOX.

[Our correspondents are reminded that prolixity is a great bar to publication, and that brevity of style and conciseness of statement greatly facilitate early insertion.]

### CHEYNE HOSPITAL FOR SICK AND INCURABLE CHILDREN, CHEYNE WALK, CHELSEA.

Sir,—The work of the Cheyne Hospital is somewhat unique. It only accepts cases which are refused or discharged by general hospitals, and it receives children for an indefinite period averaging over two years, frequently extending to three or four times that period. Of these technically "incurable" cases many, of course, are really hopeless. In these, suffering and discomfort can always be, to some extent, relieved by constant attention. The children remain here in peace and quietude till their end comes, and the homes and parents are released from the terrible incubus of a sick child requiring continual care to provide for the needs of the happier and healthier members of the family.

But many of these cases are only "incurable" because general hospitals cannot possibly devote to them the space and time required to successfully combat deep-seated disease. Here they are treated under the best conditions of nursing and ventilation uninterruptedly for years if necessary; and during the past fourteen years over two-thirds of the children admitted here have eventually been discharged either cured or, more or less, permanently relieved.

The capacity of the hospital was recently more than doubled, and its fifty beds are always full. Funds are much needed to meet the regular current expenditure, donations having fallen off very considerably last year; indeed it is difficult to enlist for the very slow and quiet work of an institution like this, the widespread sympathy and support, readily accorded to general children's hospitals at this season.

Reginald Blunt, Secretary.

